# Physical Activity Level, Mediterranean Diet Adherence, and Emotional Intelligence as a Function of Family Functioning in Elementary School Students

**DOI:** 10.3390/children8010006

**Published:** 2020-12-24

**Authors:** Eduardo Melguizo-Ibáñez, Virginia Viciana-Garófano, Félix Zurita-Ortega, José Luis Ubago-Jiménez, Gabriel González-Valero

**Affiliations:** Department of Didactics of Musical, Plastic and Corporal Expression, University of Granada, 18071 Granada, Spain; edumeliba@correo.ugr.es (E.M.-I.); vviciana@ugr.es (V.V.-G.); felixzo@ugr.es (F.Z.-O.); ggvalero@ugr.es (G.G.-V.)

**Keywords:** family function, Mediterranean diet, emotional intelligence, physical activity

## Abstract

(1) Background: Family is considered as one of the most important elements for the transmission of healthy habits that improve the lives of students. For this reason, the present study aims to describe the degree of family functionality, emotional intelligence, Mediterranean diet adherence, and extra-curricular physical activity engagement. A further aim is to perform a correlational analysis between these variables. (2) Methods: To this end, an ad hoc questionnaire was used, alongside the APGAR, KIDMED, and Trait Meta Mood Scale (TMMS-24). (3) Results: Finally, the data suggest that a high percentage of students need to improve their diet. Further, students reporting severe family dysfunction showed worse outcomes. Thus, levels of emotional clarity were lower when family functionality was poor. Poor diet quality was also associated with lower emotional attention, with Mediterranean diet adherence being positively related to emotional clarity and repair, as well as normal family functionality. (4) Conclusions: Boys showed higher levels of adherence to the Mediterranean diet adherence, while girls reported higher family functionality. Thus, compliance with the minimum recommendations for physical activity engagement was associated with adequate adherence to the Mediterranean diet. The importance of diet for obtaining an optimal physical condition, adequate emotional state, and family functionality is highlighted.

## 1. Introduction

Currently, the family context is conceived to be one of the most important elements with regards to whether students acquire appropriate daily life habits [1,2]. For this reason, the family ambit is assumed to be a vital determinant of human behaviour [3,4]. Family functioning directly influences the transmission of positive habits, in addition to impacting upon the way in which primary school students are raised by their parents [5,6]. It can lead to negative parental behaviours, which will impair later positive development of skills in members making up the family nucleus [6,7,8,9]. These types of behaviours directly impact upon primary students. Implications include poor academic performance, negative behaviour towards peers, increased likelihood of suffering nutritional diseases, and inappropriate emotional development [10,11,12,13].

Emotions represent a fundamental factor in the development of the person [14,15]. This term can be defined as a set of interactions that take place between objective and subjective factors. These factors pertain to a neuronal-hormonal means of producing affective experiences and are manifested in determined behaviours [16,17,18]. The appropriate use of emotions provokes a state of positive wellbeing in elementary students as it attributes them with sufficient skills and resources to overcome adverse situations [19,20,21]. Being emotionally intelligent implies having control over emotions and being able to use them effectively. The concept of emotional intelligence emerges from this idea. This concept can be defined as the innate ability of an individual to take what they perceive, and understand and manipulate these perceptions from both their own emotions and those of others. The aim of this is to function in society in an intelligent and appropriate way [16,17,18,22].

Likewise, a number of studies indicate that appropriate emotional development is positively related with following a healthy diet [23,24,25]. The Mediterranean diet is characterised by possessing a high number of carbohydrate-rich foods. Examples of such foods include legumes, nuts, fruits, and vegetables. It also incorporates vegetable fats, such as olive oil, and prioritises the consumption of fish over red meats [26,27,28]. Simultaneously, this diet provides a series of health benefits such as improved blood pressure, attenuated glucose concentration in the blood, reduced likelihood of suffering from diseases that are cardiovascular in nature, and reduced likelihood of suffering from cancer. It is also related with a higher life expectancy and correct biological development [29,30,31,32]. Further, following a healthy diet is also positively related with a potentially reduced risk of infection and symptom severity in relation to Coronavirus Disease 2019 (COVID-19) [33,34].

Finally, increasingly sedentary lifestyles are drastically reducing the amount of extra-curricular physical activity engaged in by primary school students [35,36]. Physical activity offers a number of benefits to individuals. Such benefits can be psychological in nature, such as through improved mood states and reduced stress. On the other hand, benefits may be social in nature as physical activity promotes sociability and increases social integration and autonomy [37,38,39].

Thus, the main objective of the present research is to analyse and study levels of family disfunction, Mediterranean diet adherence, emotional intelligence, and physical activity engagement in primary school students. A further aim is to establish existing relationships between Mediterranean diet adherence, emotional intelligence, physical activity engagement, and family disfunction in elementary school students.

## 2. Materials and Methods

### 2.1. Design and Participants

A non-experimental study was carried out that was descriptive and cross-sectional in nature. The sample was formed by 189 students from the province of Granada with self-reported ages between 11 and 12 years (*M* = 11.45 ± 0.31). Sample distribution was homogenous, with 101 (53.4%) being male and 88 (46.6%) being female. Convenience sampling was used, inviting students to participate who were undertaking the final year of the primary education school stage.

### 2.2. Instruments

An ad-hoc questionnaire was used for data collection, which was designed to collect sociodemographic variables such as sex and age. It also estimated the level of physical activity engaged in outside of timetabled school hours. Responses were categorised as “yes” or “no” according to whether they met the recommended physical activity levels proposed by the World Health Organization [40].

In order to collect data in relation to the variable of emotional intelligence, the Trait Meta-Mood Scale (TMMS-24) was used, which was conceived and developed by Salovey et al. [41]. The present research study used the version adapted into Spanish by Fernández-Berrocal et al. [42]. The aforementioned instrument is comprised of a total of 24 items evaluated along a five-point Likert type scale (from 1 = “disagree” to 5 = “completely agree”). The first eight items are summed to produce a score for emotional attention (AE). The sum of the following eight items is used to evaluate emotional clarity (CE) and, finally, the sum of the final eight items reflects emotional repair (RE). Reliability of the overall set of these items in the present study was α = 0.893. Similar values were obtained for the corresponding indices pertaining to AE (α = 0.802), CE (α = 0.845), and RE (α = 0.791). These outcomes suggest that the data obtained were reliable.

In order to evaluate the type of diet followed by participants, the version of the KIDMED test adapted into Spanish by Serrá-Majem et al. [43] was used. This questionnaire is formed by a total of 16 questions that can be responded to in a negative or positive way. Items 5, 11, 13, and 15 are negative in nature with positive affirmations indicating worse indices. They are, therefore, negatively scored (−1 point). The remaining questions reflect positive responses, with agreement being scored with +1 points. Negative responses to these questions do not reflect any type of score and thus are given a zero-sum value (0). Final scores for this tool range between −4 and 12 points. Values are categorised according to three groups of Mediterranean diet adherence as a function of the obtained score. The groupings are as follows: optimal diet (≥8 points), needs improvement (2–7 points), and low-quality diet (≤1). Reliability analysis of the data obtained for this questionnaire produced acceptable results (α = 0.671).

Finally, in order to collect data relating to the variable describing family functioning, the APGAR questionnaire elaborated by Austin and Huberty [44] was used. Specifically, the present study used the version adapted into Spanish by Suarez and Alcalá [45]. This questionnaire includes a total of five questions about the relationship maintained between students and their family. In order to obtain the final score, the scores provided in relation to each value indicated on a three-point Likert type scale are considered. In this sense, 0 relates to “almost never”, 1 equates to “sometimes”, and 2 describes “almost always”. Finally, all of the questions are summed together in order to obtain a final score. The reliability of the data reported in the present paper was α = 0.760.

### 2.3. Procedure

In order to complete the data collection processes, the different schools selected via convenience sampling were contacted. Once permission was received from the schools, an information pack was developed that was targeted towards students’ legal guardians so that students could participate in the study while ensuring anonymity at all times. Researchers were present throughout the data collection processes in order to resolve any doubts arising during questionnaire completion. The present study complied with the ethical principles for research with human subjects established by the Declaration of Helsinki in 1975. All processes were also conducted under the supervision of the research ethics committee at the University of Granada (1230/CEIH/2020).

### 2.4. Data Analysis

All data were analysed using the statistical program SPSS 25.0 (SPSS, IBM, SPSS Statistics, v.25.0, Chicago, IL, USA). The normality of the data and homogeneity of the variables were examined according to the Kolmogorov–Smirnov test. Following this, descriptive analysis was conducted via an analysis of frequencies and means. For the comparative analysis, contingency tables and Student’s *t*-test for independent samples were used. Differences between participants were determined via Pearson chi-square analysis. One-way analysis of variance (ANOVA) comprising a single factor with Bonferroni post-hoc analysis was also used to conduct between-variable analysis. Likewise, bivariate Pearson correlations were conducted. The level of significance was set at *p* < 0.05 and *p* < 0.01. The magnitude of differences (effect size; ES) was obtained according to standardised measures of Cohen’s d [46]. This value is interpreted as being null (0–0.19), small (0.20–0.49), medium (0.50–0.79), or large (≥0.80) [47]. Finally, 95% confidence intervals (95% CI) were calculated for each effect size.

## 3. Results

The results obtained reveal that a total of 53.4% (*n* = 101) of the sample was male and 46.6% (*n* = 88) was female. Moving on to the type of family functioning found, it was observed that 89.9% (*n* = 170) of participants exhibited normal functioning; followed by 9.0% (*n* = 17) who showed moderate functioning; and, finally, 1.1% (*n* = 2) who reported serious dysfunction. When observing data pertaining to Mediterranean diet adherence, it is highlighted that 58.2% (*n* = 110) of students reported dietary habits that needed some improvement; followed by 31.2%, *n* = 59) who reported optimal dietary patterns; and, finally, 10.6% (*n* = 20) who followed an extremely low-quality diet (Table 1). The sample reported values that suggested that most individuals were highly physically active, with 87.8% (*n* = 166) engaging in extra-curricular physical activity, while only 12.2% (*n* = 23) reported not engaging in this behaviour. Shifting our focus to emotional attention, it is observed that 62.4% (*n* = 118) pay appropriate attention to their emotions; followed by 19.0% (*n* = 36) who pay little attention; and, finally, 18.5% (*n* = 35) who pay too much attention to their emotions. Moving on to emotional clarity, 57.7% (*n* = 109) reported scores that reflect an appropriate level; followed by 24.9% (*n* = 47) whose values reflect a need to improve emotional clarity; and, finally, 17.5% (*n* = 33) who demonstrate excellent emotional clarity. Finally, 59.8% (*n* = 113) demonstrated appropriate levels of emotional repair; followed by 24.3% (*n* = 46) who exhibited an excellent level; and, finally, 15.9% (*n* = 30) whose values reflected a need for improvement.

Table 2 presents basic descriptive statistics expressed as means, alongside outcomes of the correlational analysis. The values obtained for Mediterranean diet adherence confirm the general need to improve these dietary patterns (*M* = 6.32 ± 2.28). Statistically significant associations were produced with the variables of emotional attention (IEAE) (*r* = 0.199 **), emotional clarity (IECE) (*r* = 0.207 **), and emotional repair (IERE) (*r* = 0.222 **). These reveal that following an optimal diet is directly related with excellent indices of emotional clarity and repair, although excessive emotional attention was also related with this trait. In this way, diet was positively related with family functioning (*r* = 0.191 **), showing that, when family functioning is normal, participants tend to present optimal Mediterranean dietary patterns. Associations between the dimensions of emotional intelligence produced the highest values with regards to IERE (*M* = 3.74 ± 0.81), followed by IECE (*M* = 3.58 ± 0.85) and IEAE (*M* = 3.48 ± 0.80). In this sense, the IEAE was indirectly associated with family functioning (*r* = −0.131 *), while the IECE was indirectly related with this variable (*r* = 0.168 *). Paying too much attention to emotions was related with moderate-serious family dysfunction, while appropriate emotional clarity was associated with normal family functioning.

The outcomes of relational analysis of the variable of family functioning are found in Table 3. The frequency analysis did not produce any statistically significant differences (*p* ≥ 0.05). Nonetheless, when examining the relationships produced between the mean values recorded by students with serious family dysfunction, lower levels of adherence to the Mediterranean diet (*M* = 2.50 ± 2.12) were reported in comparison with students with moderate dysfunction (*M* = 6.34 ± 2.25; *d* = 1.712) or normal functioning (*M* = 6.58 ± 2.31; *d* = 1.767). The same trend also emerged with regards to emotional clarity, with lower scores being seen among those reporting serious emotional dysfunction (*M* = 2.62 ± 0.23), relative to those with moderate (*M* = 3.59 ± 0.85; *d* = 1.174) or normal functioning (*M* = 3.61 ± 0.89; *d* = 1.115). A large effect size was found with regards to the relationship produced between these study variables (*d* ≥ 0.800).

Table 4 presents the relational study pertaining to the variable describing Mediterranean diet adherence, with this analysis producing statistically significant differences (*p* ≤ 0.05). It can be seen that those students who report diets in need of improvement and appropriate emotional clarity (61.8%; *n* = 68) outnumber those who follow diets that require improvement and report excellent levels of emotional clarity (14.5%; *n* = 16). At the same time, it can also be observed that the number of students who follow poor quality diets and present appropriate levels of emotional repair (70.0%; *n* = 14) is higher than the number of those who consume a low-quality diet and demonstrate levels of emotional repair that need improvement (25.0%; *n* = 5). Along these lines, it serves to highlight that participants with a low-quality diet obtained lower scores on the IEAE (*M* = 3.05 ± 0.76), in comparison with those with an optimal diet (*M* = 3.59 ± 0.80; *d* = 0.683). The same pattern was seen in relation to IECE (*M* = 3.14 ± 0.82 vs. *M* = 3.72 ± 0.90; *d* = 0.658), IERE (*M* = 3.34 ± 0.74 vs. *M* = 3.86 ± 0.82; *d* = 0.649), and APGAR (*M* = 1.61 ± 0.37 vs. *M* = 1.79 ± 0.27; *d* = 0.604) scores. A medium effect size was found for the relationship between these studied variables (*d* ≥ 0.600).

Table 5 presents the relational study of frequencies pertaining to sex, for which statistically significant differences were found (*p* ≥ 0.05). Nonetheless, examination of the means recorded reveals that males presented higher values for Mediterranean diet adherence (*M* = 6.96 ± 2.45), producing a significant, albeit small, effect size (*p* = 0.044; *d* = 0.355). Further, females presented higher levels of family functioning (*M* = 1.97 ± 0.29), with a small effect size being produced (*p* = 0.039; *d* = 0.441).

Table 6 presents the outcomes of the relational study conducted between the physical activity engagement variable and other study variables, with no statistically significant differences (*p* ≥ 0.05) being found between frequencies. In contrast, it was demonstrated that participants who met minimum physical activity recommendations presented better indices with regards to Mediterranean diet adherence (*M* = 6.95 ± 2.09; *d* = 0.412) and IERE (*M* = 3.79 ± 0.80; *d* = 0.461). In this case, although significant outcomes were produced with regards to effect size (*p* ≤ 0.05), the magnitude of this effect size was small.

## 4. Discussion

The relationship between the family nucleus and adherence to patterns that are beneficial to health appear to have a hugely important role when it comes to avoiding diseases with a cardiovascular or mental origin. The aim of the present study was to describe and correlate levels of family functioning, Mediterranean diet adherence, emotional intelligence, and physical activity engagement in primary school students. This objective is related to previously conducted studies that were similar in nature, such as those conducted by Muros et al. [23], Cheung [48], and Hemmingsson [8]. The importance of this study lies in examining the incidence of Mediterranean diet adherence in the context of the extent of family functioning, emotional skill development, and levels of physical activity engagement.

With regards to following a healthy diet, it is observed that more than half of the sample requires improvement. These data are hugely contrasting to those uncovered by Canto et al. [49]. In contrast, this prior study concluded that students generally reported high levels of Mediterranean diet adherence. Nonetheless, the results of the present research study are largely similar to those concluded by Asensi et al. [50], who established that following a positive diet was related to the area in which students carried out their day-to-day activities. These outcomes may be explained by the reasons put forth by Galán-López et al. [51] and Rosi et al. [28], who explain that students older than eleven years start to take control over their own diet, in this way increasing their negative dietary patterns. In addition, El Mokhatari et al. [52] concluded that worse dietary outcomes are obtained at the educational stage corresponding to compulsory secondary education, with this stage also coinciding with the start of adolescence.

Moving on to consider levels of physical activity engagement, it was revealed that more than two thirds of the population was physically active. This coincides with that reported by Mitchell and Steele [53] and Tyler et al. [54], who found that the majority of the population is physically active and largely avoids sedentary lifestyle habits. Grasten et al. [55], Groffik et al. [56], Petrie et al. [57], and Reid et al. [58] argue that physical activity engagement outside of school hours is encouraged by positive attitudes experienced in physical activity classes. In this sense, this prior research also outlined that the majority of students held positive attitudes towards the aforementioned subject.

In the emotional context, it was observed in the three examined emotional variables that more than half of the sample reflected adequate levels. This suggests that students maintain sufficient control over aspects demanding emotional attention, clarity and repair. These outcomes are similar to those achieved by Jauk and Ehrenthal [59] and Núñez and Muñoz [60], who produced evidence that students undertaking primary education exhibit appropriate emotional control.

In the section pertaining to the extent of family functioning, it was observed that the majority of students reported normal functioning; followed by those reporting moderate dysfunction; and, finally, those reporting very high levels of family dysfunction. Nie et al. [9] and Kim et al. [61] unveiled largely similar results to those of the present study, with more than two thirds of their overall samples reflecting normal family functioning.

In consideration of the degree of family functioning, it is evident that students who present a normal level of family functioning reflect better adherence to healthy dietary patterns than those who report moderate dysfunction. These results are largely similar to those produced by Braden [62], Leclerc et al. [63], and Menghetti et al. [64], with these authors also highlighting that a large degree of family dysfunction is related to following a healthy diet and leading a healthy lifestyle.

Moving on to physical activity engagement, it is presented here that students with normal family functioning are more likely to engage in physical activity, whereas students who experience moderate dysfunction present worse indices of extra-curricular physical activity engagement. This corroborates the findings reported by Dinkel et al. [65]. In contrast, Cheung [48], Langlois et al. [66], Yang-Huang et al. [67], and Yoong et al. [68] propose that families with a higher educational level and socioeconomic status are more aware of the health benefits of engaging in physical activity for their children. With regards to levels of emotional attention, it can be seen that students who report a normal degree of family functioning report more regular values in respect to the three levels of emotional functioning. On the other hand, students who exhibit a moderate or severe degree of dysfunction show impairments in this aspect. These results are again consistent with other research studies, for example, those reported by Szczesniak and Tulecka [69], Trigueros et al. [70], and Weinzimmer et al. [71]. Specifically, these prior studies highlighted that more emotionally stable homes are more effective when it comes to transmitting and teaching children how to use and control emotions appropriately.

Moving on to the analysis involving the variable pertaining to sex, it can be seen that the majority of the population is physically active. These outcomes are in accordance with those acquired by Deutsch et al. [72] and Kuritz et al. [73], who revealed that more than two thirds of the population engages in physical activity outside of school hours. Further, it can also be observed that females are less physically active than males when it comes to engaging outside of the school timetable. This supports the conclusions stated by Gaylis et al. [74] and Labrador [75], who established that females tended to abandon physical activity at early ages than males. Proceeding to consider the type of diet followed, it is seen that males reported more positive indices than females in that they tended to follow healthier diets. This outcome coincides with data obtained by Bonaccorsi et al. [76], Labrador [75], and Kouvari et al. [77], who argued that sex is a key factor when it comes to following an energy balanced diet or a diet that is beneficial to health. With regards to levels of emotional intelligence, the present study reveals that females exhibit better outcomes for the emotional variables than males. Similarly, Bliton et al. [78] and Miller [79] also emphasised that more than two thirds of the male population needs to engage in specific programs to improve various emotional indices.

We now consider the relational study pertaining to physical activity engagement. Coen et al. [80], Han [81], and Kruk et al. [82] highlighted in their various studies that participants who regularly engaged in physical activity demonstrated better indices of emotional control and perception, while also promoting socialisation and improved mood states.

With regards to the relational analysis of Mediterranean diet adherence and physical activity engagement, it can be seen that, regardless of the type of diet followed, the majority of the sample was physically active. These data do not agree with the results presented by Barnes et al. [83] and Laxmaiah et al. [84], who established that physical activity engagement is positively associated with the consumption of an energy-balanced diet. On the other hand, Hardman et al. [85] and Jospe et al. [86] argue that calorie consumption will depend on the type of physical activity in question.

Finally, statistically significant differences were found when associating Mediterranean diet adherence and emotional clarity variables. In this sense, it could be observed that more students consume a diet that requires improvement and demonstrate appropriate emotional clarity than those who demonstrate excellent clarity and consume a diet requiring improvement. At the same time, it can also be observed that the number of students who consume a low-quality diet and an appropriate level of emotional repair was higher than those consuming a poor diet and demonstrating emotional indices in need of improvement. Largely similar results were achieved by Chang et al. [87] and Jin et al. [88]. These authors argued that, when some students experience unpleasant emotions, they tend to experience variations in their dietary patterns and over-feeding. Such practices are used to improve the mood state of individuals.

## 5. Conclusions

In consideration of the descriptive study, it emerged that the majority of the sample experienced normal levels of family functioning. At the same time, close to two thirds of the overall sample needed to improve their diet, more than three quarters of students were physically active and individuals typically reported appropriate levels of three components of the emotional variable. 

It was highlighted that students who exhibited a severe degree of dysfunction produced worse outcomes in relation to a healthy diet in comparison with students with normal or excellent family functioning. Likewise, lower levels of emotional clarity were also uncovered in students with poor levels of family functioning.

Shifting attention to the type of diet followed, it is observed that participants with low-quality diets obtained lower emotional attention indices relative to those who consumed diets that were optimal in nature. Likewise, it was also uncovered that following an optimal diet was positively related with appropriate levels of emotional clarity and repair, in addition to excellent family functioning.

Moving on to sex, it can be concluded that males evidence better outcomes with regards to following a healthy diet, while females evidence better outcomes with regards to family functioning.

Finally, in consideration of levels of physical activity engagement, it is shown that participants who met physical activity recommendations evidenced greater adherence to a Mediterranean diet.

### Limitations and Future Perspectives

The main limitation to highlight is that the present study is cross-sectional and performed only a single measurement in one population at a specific time-point. This only permits relationships between variables to be identified at this aforementioned time-point and does not permit causal relationships to be examined over a determined time period. 

Another limitation is that the sample was composed of primary school students from a highly specific geographical area. This prevents outcomes from being generalised to broader geographical areas pertaining to national or autonomous regions.

Regarding future perspectives, it would be really interesting study how strategies such as mindfulness influence healthy habits. The study of Soriano et al. [89] shows how mindfulness helps people to have better lifestyles; thus, concerning this study, it would be interesting to design a program for children based on how mindfulness can help children follow healthy habits.

## Figures and Tables

**Table 1:** Basic descriptive frequencies of the study.

	Frequency (*n*)	Percentage (%)		Frequency (*n*)	Percentage (%)
	Sex			Emotional attention	
**Male**	101	53.4%	**Little**	36	19.0%
**Female**	88	46.6%	**Appropriate**	118	62.4%
	**Family functioning**		**Too much**	35	18.5%
**Normal functioning**	170	89.9%		**Emotional clarity**	
**Moderate dysfunction**	17	9.0%	**Needs improvement**	47	24.9%
**Serious dysfunction**	2	1.1%	**Appropriate**	109	57.7%
	**Mediterranean diet adherence**		**Excellent**	33	17.5%
**Optimal diet**	59	31.2%		**Emotional repair**	
**Needs improvement**	110	58.2%	**Needs improvement**	30	15.9%
**Low quality**	20	10.6%	**Appropriate**	113	59.8%
	**Physical activity engagement**		**Excellent**	46	24.3%
**Does not engage**	23	12.2%			
**Engages**	166	87.8%			

**Table 2:** Descriptive means outcomes of the correlational analysis of study variables.

Variables	M	SD	Minimum	Maximum	(1)	(2)	(3)	(4)	(5)
**KIDMEzD** **(1)**	6.32	2.28	1.00	11.00	1	0.199 **	0.207 **	0.222 **	0.191 **
**IEAE** **(2)**	3.48	0.80	1.25	5.00		1	0.480 **	0.443 **	−0.131 *
**IECE** **(3)**	3.58	0.85	1.50	5.00			1	0.576 **	0.168 *
**IERE** **(4)**	3.74	0.81	1.50	5.00				1	0.010
**APGAR** **(5)**	1.75	0.32	0.60	2.00					1

**Note**. Mediterranean diet adherence (KIDMED); emotional attention (IEAE); emotional clarity (IECE); emotional repair (IERE); family functioning (APGAR). ** *p* < 0.01 * *p* < 0.05.

**Table 3:** Relational analysis of the variable describing family functioning.

Variable	Category	Family Functioning					Sig.
		Normal		Moderate	Severe		
**KIDMED**	Optimal diet	31.2%; *n* = 53		35.3%; *n* = 6	0.0%; *n* = 0		*p* = 0.402
	Needs improvement	58.2%; *n* = 99		58.8%; *n* = 10	50.0%; *n* = 1		
	Low quality	10.6%; *n* = 18		5.9%; *n* = 1	50.0%; *n* = 1		
**IEAE**	Lacks attention	19.4%; *n* = 33		11.8%; *n* = 2	50.0%; *n* = 1		*p* = 0.561
	Appropriate	61.2%; *n* = 104		76.5%; *n* = 13	50.0%; *n* = 1		
	Too much	19.4%; *n* = 33		11.8%; *n* = 2	0.0%; *n* = 0		
**IECE**	Needs improvement	24.1%; *n* = 41		23.5%; *n* = 4	100.0%; *n* = 2		*p* = 0.191
	Appropriate	58.2%; *n* = 99		58.8%; *n* = 10	0.0%; *n* = 0		
	Excellent	17.6%; *n* = 30		17.6%; *n* = 3	0.0%; *n* = 0		
**IERE**	Needs improvement	15.3%; *n* = 26		17.6%; *n* = 3	50.0%; *n* = 1		*p* = 0.261
	Appropriate	61.8%; *n* = 105		41.2%; *n* = 7	50.0%; *n* = 1		
	Excellent	22.9%; *n* = 39		41.2%; *n* = 7	0.0%; *n* = 0		
**PA**	Does not engage	11.8%; *n* = 20		17.6%; *n* = 3	0.0%; *n* = 0		*p* = 0.677
	Engages	88.2%; *n* = 150		82.4%; *n* = 14	100.0%; *n* = 2		
**Variable**	**Family functioning**	**Mean**	**SD**	**F**	**Sig.**	**ES (d)**	**95% CI**
**KIDMED**	Normal	6.58	2.31	3.981	*p* ≤ 0.05 ^b,c^	1.767 ^b^ 1.712 ^c^	[0.361; 3.174] ^b^ [0.149; 3.275] ^c^
	Moderate	6.34	2.25				
	Severe	2.50	2.12				
**IEAE**	Normal	3.47	0.82	0.887	*p* ≥ 0.05	NP	NP
	Moderate	3.68	0.67				
	Severe	3.00	0.35				
**IECE**	Normal	3.61	0.89	2.287	*p* ≤ 0.05 ^b,c^	1.115 ^b^ 1.174 ^c^	[−0.284; 2.514] ^b^ [−0.338; 2.686] ^c^
	Moderate	3.59	0.85				
	Severe	2.62	0.23				
**IERE**	Normal	3.74	0.79	1.102	*p* ≥ 0.05	NP	NP
	Moderate	3.88	1.03				
	Severe	3.00	0.17				

**Note 1.** Mediterranean diet (KIDMED); emotional attention (IEAE); emotional clarity (IECE); emotional repair (IERE); family functioning (APGAR); physical activity (PA). **Note 2.** Differences between normal family functioning and severe dysfunction (^b^); differences between moderate and serious family dysfunction (^c^). ES, effect size; CI, confidence interval.

**Table 4:** Relational study pertaining to the Mediterranean diet adherence variable.

Variable	Category	Mediterranean Diet					Sig.
		Optimal Diet	Needs Improvement		Low Quality		
**IEAE**	Lacks attention	13.6%; *n* = 8	19.1%; *n* = 21		35.0%; *n* = 7		*p* = 0.094
	Appropriate attention	64.4%; *n* = 38	60.9%; *n* = 67		65.0; *n* = 13		
	Too much attention	22.0%; *n* = 13	20.0%; *n* = 22		0.0%; *n* = 0		
**IECE**	Needs improvement	20.3%; *n* = 12	23.6%; *n* = 26		45.0%; *n* = 9		*p* = 0.039
	Appropriate	52.5%; *n* = 31	61.8%; *n* = 68		50.0%; *n* = 10		
	Excellent	27.1%; *n* = 16	14.5%; *n* = 16		5.0%; *n* = 1		
**IERE**	Needs improvement	13.6%; *n* = 8	15.5%; *n* = 17		25.0%; *n* = 5		*p* = 0.163
	Appropriate	54.2%, *n* = 32	60.9%, *n* = 67		70.0%; *n* = 14		
	Excellent	32.2%; *n* = 19	23.6%; *n* = 26		5.0%; *n* = 1		
**AF**	Does not engage	10.2%; *n* = 6	13.6%; *n* = 15		10.0%; *n* = 2		*p* = 0.767
	Engage	89.8%; *n* = 53	86.4%; *n* = 95		90.0%; *n* = 20		
**Variable**	**Diet**	**Mean**	**SD**	**F**	**Sig.**	**ES (d)**	**95% CI**
**IEAE**	Optimal	3.59	0.80	3.599	*p* ≤ 0.05 ^a^	0.683 ^a^	[0.165; 1.201] ^a^
	Needs improvement	3.50	0.80				
	Low quality	3.05	0.76				
**IECE**	Optimal	3.72	0.90	3.578	*p* ≤ 0.05 ^a^	0.658 ^a^	[0.141; 1.176] ^a^
	Needs improvement	3.59	0.81				
	Low quality	3.14	0.82				
**IERE**	Optimal	3.86	0.82	3.152	*p* ≤ 0.05 ^a^	0.649 ^a^	[0.132; 1.166] ^a^
	Needs improvement	3.75	0.80				
	Low quality	3.34	0.74				
**APGAR**	Optimal	1.79	0.27	2.792	*p* ≤ 0.05 ^a^	0.604 ^a^	[0.089; 1.120] ^a^
	Needs improvement	1.73	0.33				
	Low quality	1.61	0.37				

**Note 1.** Emotional attention (IEAE); emotional clarity (IECE); emotional repair (IERE); physical activity (PA); family functioning (APGAR). **Note 2.** Differences between a diet that needs improvement and a low-quality diet (^a^).

**Table 5:** Relational study according to the sex of participants.

Variable	Category	Sex								Sig.	
		Male				Female					
**AF**	Doesn’t engage	9.9%; *n* = 10				14.8%; *n* = 13				*p* = 0.307	
	Engage	90.1%; *n* = 91				85.2%; *n* = 75					
**KIDMED**	Optimal diet	35.6%; *n* = 36				26.1%; *n* = 23				*p* = 0.128	
	Needs improvement	51.5%; *n* = 52				65.9%; *n* = 58					
	Low quality	12.9%; *n* = 13				8.0%; *n* = 7					
**IEAE**	Lacks attention	19.8%; *n* = 20				18.2%; *n* = 16				*p* = 0.807	
	Appropriate	63.4%; *n* = 64				61.4%; *n* = 54					
	Too much	16.8%; *n* = 17				20.5%; *n* = 18					
**IECE**	Needs improvement	25.7%; *n* = 26				23.9%; *n* = 21				*p* = 0.932	
	Appropriate	56.4%; *n* = 57				59.1%; *n* = 52					
	Excellent	17.8%; *n* = 18				17.0%; *n* = 15					
**IERE**	Needs improvement	17.8%; *n* = 18				13.6%; *n* = 12				*p* = 0.735	
	Appropriate	58.4%; *n* = 59				61.4%; *n* = 54					
	Excellent	23.8%; *n* = 24				25.0%; *n* = 22					
**Variable**	**Sex**			**Levene Test**			***t*-Test**		**ES (d)**		**95% CI**
		**M**	**SD**	**F**	**Sig.**		**t**	**Sig.**			
**KIDMED**	M	6.96	2.45	2.960	0.087		0.919	0.044	0.355		[0.067; 0.643]
	F	6.15	2.07								
**IEAE**	M	3.42	0.83	0.183	0.669		−1.146	0.253	NP		NP
	F	3.55	0.78								
**IECE**	M	3.55	0.84	0.091	0.763		−0.566	0.572	NP		NP
	F	3.62	0.87								
**IERE**	M	3.71	0.82	0.3450	0.558		−0.644	0.521	NP		NP
	F	3.78	0.80								
**APGAR**	M	1.73	0.34	3.359	0.068		−0.855	0.039	0.441		[0.151; 0.730]
	F	1.97	0.29								

**Note.** Physical activity (PA); Mediterranean diet adherence (KIDMED); emotional attention (IEAE); emotional clarity (IECE); emotional repair (IERE); family functioning (APGAR); male gender (M); female gender (F).

**Table 6:** Relational study pertaining to the variable of physical activity engagement.

Variable	Category	PA							Sig.
		No			Yes				
**IEAE**	Lacks attention	13.0%; *n* = 3			19.9%; *n* = 33				*p* = 0.479
	Appropriate	73.9%; *n* = 17			60.8%; *n* = 101				
	Too much	13.0%; *n* = 3			19.3%; *n* = 32				
**IECE**	Needs improvement	30.4%; *n* = 7			24.1%; *n* = 40				*p* = 0.793
	Appropriate	52.2%; *n* = 12			58.4%; *n* = 97				
	Excellent	17.4%; *n* = 4			17.5%; *n* = 29				
**IERE**	Needs improvement	21.7%; *n* = 5			15.1%; *n* = 25				*p* = 0.579
	Appropriate	60.9%; *n* = 14			59.6%; *n* = 99				
	Excellent	17.4%; *n* = 4			25.3%; *n* = 42				
**Variable**	**PA**			**Levene Test**		***t*-Test**		**ES (d)**	**95% CI**
		**M**	**SD**	**F**	**Sig.**	**t**	**Sig.**		
**KIDMED**	No	6.08	2.25	0.958	0.810	−0.527	0.042	0.412	[−0.026; 0.850]
	Yes	6.95	2.09						
**IEAE**	No	3.42	0.83	0.116	0.733	−0.401	0.689	NP	NP
	Yes	3.49	0.80						
**IECE**	No	3.42	0.89	0.003	0.957	−0.958	0.339	NP	NP
	Yes	3.61	0.85						
**IERE**	No	3.42	0.82	1.252	0.616	−2.045	0.039	0.461	[0.023; 0.900]
	Yes	3.79	0.80						
**APGAR**	No	1.78	0.29	0.045	0.832	0.461	0.646	NP	NP
	Yes	1.74	0.32						

**Note.** Physical activity (PA); Mediterranean diet adherence (KIDMED); emotional attention (IEAE); emotional clarity (IECE); emotional repair (IERE); family functioning (APGAR); male gender (M).

## References

[1] Pain E. (2020). Is Teen Risk of Having Sex with Strangers Associated with Family Environment? Family Processes, Household Structure and Adolescent Sex with Strangers. Youth Soc..

[2] Holzinger D., Dall M., Sanduvete-Chaves S., Saldana D., Chacón-Moscoso S., Fellinger J. (2020). The Impact of Family Environment of Language Development of Children with Cochlear Implants: A systematic Review and Meta-Analysis. Ear Hear..

[3] Wenden E.J., Lester L., Zubrick S.R., Ng M., Christian H.E. (2020). The relationship between dog ownership, dog play, family dog walking, and pre-schooler social- emotional development: Findings from the PLAYCE observational study. Pediatr. Res..

[4] Woods T. (2020). Rural grandparenting through a family development lens: Implications for social work practice. J. Soc. Work Prac..

[5] Tan J.H.P., Conlon C., Tsamparli A., O’Neill D., Adamis D. (2019). The association between family dysfunction and admission to an acute mental health inpatient unit: A prospective study. Ir. J. Psychol. Med..

[6] Wang Y., Tian L., Guo L., Huebner E.S. (2020). Family dysfunction and Adolescents’ anxiety and depression: A multiple mediation model. J. Appl. Dev. Psychol..

[7] Guo L., Tian L., Huebner E.S. (2018). Family dysfunction and anxiety in adolescents: A moderated mediation model of self-esteem and perceived school stress. J. Sch. Psychol..

[8] Hemmingsson E. (2018). Early childhood obesity risk factors: Socioeconomic adversity, family dysfunction, offspring distress, and junk food self-medication. Curr. Obes. Rep..

[9] Nie Q., Tian L., Huebner E.S. (2019). Relations among Family Dysfunction, Loneliness and Life Satisfaction in Chinese Children: A Longitudinal Mediation Model. Child. Indic. Res..

[10] Kidwwell R.E., Eddleston K.A., Kellermanss F.W. (2018). Learning bad habits across generation: How negative imprints affect human resource management in the family firm. Hum. Resour. Manag. Rev..

[11] Laghi F., McPhie M.L., Baumgartner E., Rawana J.S., Pompili S., Baiocco R. (2016). Family functioning and dysfunctional eating among Italian adolescents: The moderating role of gender. Child Psychiatry Hum. Dev..

[12] Lynch B.A., Agunwmba A., Wilson P.M., Kumar S., Jacobson R.M., Phelan S., Cristiani V., Fan C., Rutten L.J.F. (2016). Adverse family experiences and obesity in children and adolescents in the United States. Prev. Med..

[13] Ohara T., Matsuura N., Hagiuda N., Wakasugi N. (2019). The effects of correctional Education on juvenile delinquents and the factors for their overall changes: Focusing on academic performance and family-type environment. Child Fam. Soc. Work.

[14] Denervaud S., Mumenthaler C., Gentaz E., Sander D. (2020). Emotion recognition development: Preliminary evidence for and effect of school pedagogical practices. Learn. Instr..

[15] Sexton J.B., Adair K.C. (2019). Forty-five Good things: A prospective pilot study of the Hree Good Things well-being intervention in the USA for healthcare worker emotional exhaustion, depression, worklife balance and happiness. BMJ Open.

[16] Adolphs R., Mlodinow L., Barret L.F. (2019). What is an emotion?. Curr. Biol..

[17] Hoon Y., Harry H., Richards A.R. (2019). Emotional Intelligence, Unpleasant Emotions, Emotional Exhaustion, and Hob Satisfaction in Physical Education Teaching. J. Teach. Phys. Educ..

[18] Laborde S., Dosseville F., Allen M.S. (2016). Emotional intelligence in sport and exercise: A systematic review. Scand. J. Med. Sci. Sports.

[19] Jiménez-Herrera M.F., Llauradó-Serra M., Acebedo-Urdiales S., Bazo-Hernández L., Font-Jiménez I., Axelsson C. (2020). Emotions and feelings in critical and emergency caring situations: A qualitative study. BMC Nurs..

[20] Predatu R., David D.O., Meffaei A. (2020). Beliefs about emotions, negative meta-emotions, and perceived Emotional control during and emotionally salient situation in individuals with emotional disorders. Cognit. Ther. Res..

[21] Puertas-Molero P., Zurita-Ortega F., Chacón-Cuberos R., Castro-Sánchez M., Ramírez-Granizo I., González-Valero G. (2020). La inteligencia emocional en el ámbito educativo: Un meta-análisis. Ann. Physicol..

[22] Halimi F., AlShammari I., Navarro C. (2020). Emotional intelligence and academic achievement in higher education. J. Appl. Res. High. Educ..

[23] Muros J.J., Cofre-Bolados C., Arriscado D., Zurita F., Knox E. (2017). Mediterranean diet adherence is associated with lifestyle, physical fitness, and mental wellness among 10-y-olds in Chile. Nutrition.

[24] Onetti W., Álvarez-Kurogi L., Castillo-Rodríguez A. (2019). Adherence to the Mediterranean diet pattern and self-concept in adolescents. Nutr. Hosp..

[25] Tate E.B., Sprujit-Metz D., Pickering T.A., Pentz M.A. (2015). Two facets of stress and indirect effects on child diet through emotion-driven eating. Eat. Behav..

[26] Di Lellis M.A. (2020). Mediterranean Diet has a positive effect on Health. Zeits. Gastroen..

[27] González-Valero G., Ubago-Jiménez J.L., Ramírez-Granizo I.A., Puertas-Molero P. (2019). Association between motivational climate, adherence to mediterranean diet, and levels of physical activity in physical education students. Behav. Sci..

[28] Rosi A., Biasini B., Donati M., Ricci C., Scanizza F. (2020). Adherence to the Mediterranean Diet and Environmental Impact of the Diet on Primary School Children Living in Parma (Italy). Int. J. Environ. Res. Public Health.

[29] Akasheva D.U., Drapkina O.M. (2020). Mediterranenan Diet: Origin History, Main Components, Evidence of Benefits and Feasibility to Adapt to the Russian Reality. Ration. Pharmacother. Cardiol..

[30] Georgoulis M., Yiannakouris N., Kechribari I., Lamprou K., Perraki E., Vagiakis E., Kontogianni M.D. (2020). Cardiometabolic Benefits of a Weight-Loss Mediterranenan Diet/Lifestyle Intervention in Patients with Obstructive Sleep Apnea: The “MIMOSA” Randomized Clinical Trial. Nutrients.

[31] Serra-Majem L., Román-Vinas B., Sánchez-Villegas A., Guasch-Ferre M., Corella D., La Vecchia C. (2019). Benefits of the Mediterranean diet: Epidemiological and molecular aspects. Mol. Asp. Med..

[32] Tosti V., Bertozzi B., Fontana L. (2018). Health Benefits of the Mediterranean Diet: Metabolic and Molecular Mechanisms. J. Gerontol. Ser. A Biol. Sci. Med. Sci..

[33] Grant W.B., Lahore H., McDonnell S.L., Baggerly C.A., French C.B., Aliano J.L., Bhattoa H.P. (2020). Evidence that Vitamin D Supplementation Could Reduce Risk of Influenza and COVID-19 Infections and Deaths. Nutrients.

[34] Haupt-Jorgensen M., Buschard K. (2020). Can a gluten-free diet be partly protective for Covid-19 infection?. Apmis.

[35] González-Valero G., Zurita-Ortega F., Martínez-Martínez A. (2017). Motivational and physical activity panorama in students: A systematic review. ESHPA.

[36] Guldager J.D., von Seelen J., Andersen P.T., Leppin A. (2020). Do student social background and school context affect implementation of a school-based physical activity program?. Eval. Program. Plan..

[37] Callow D.D., Arnold-Nedimala N.A., Jordan L.S., Pensa G.S., Won J., Woodard J.L., Smith J.C. (2020). The Mental Health Benefits of Physical Activity in Older Adults Survive the COVID-19 Pandemic. Am. J. Geriatr. Psychiatry.

[38] Goenka S., Devarajan R. (2020). Higher Physical Activity Levels in Children Have Wide Ranging Benefits: Towards Multisectoral Action. Indian Pedia.

[39] Sfendla A., Hadrya F. (2020). Factors Associated with Psychological Distress and Physical Activity During the COVID-19 Pandemic. Health Sec..

[40] World Health Organization (2010). Recomendaciones Mundiales Sobre Actividad Física Para la Salud.

[41] Salovey P., Mayer J.D., Goldman S.L., Turvey C., Palfai T.P., Pennebaker J.W. (1995). Emotional attention, clarity, and repair: Exploring emotional intelligence using the Trait Meta-Mood Scale. Emotion, Disclosure and Health.

[42] Fernández-Berrocal P., Extremera N., Ramos N. (2004). Validity and reliability of the Spanish modifiel versin of the Trait Meta-Mood Scale. Psychol. Rep..

[43] Serra-Majem L., Ribas L., Ngo J., Ortega R.M., García A., Pérez-Rodrigo C., Aranceta J. (2004). Food, youth and the Mediterranean diet in Spain. Development of KIDMED, Mediterranean diet quality index in children and adolescents. Public Health Nutr..

[44] Austin J.K., Huberty Y.J. (1989). Revision of the family APGAR for use by 8-years old. Fam. Syst. Med..

[45] Suárez M., Alcalá M. (2014). Apgar familiar: Una herramienta para detectar disfunción familiar. Rev. Med. Paz.

[46] Cohen J. (1988). Statistical Power Analysis for the Behavioral Sciences.

[47] Cohen J. (1992). A power primer. Psychol. Bull..

[48] Cheung P.P.Y. (2017). Children’s after-school physical activity participation in Hong Kong: Does family socioeconomic status matter?. Health Educ. J..

[49] Canto E.G., López P.J.C., Guillamón A.R. (2019). Analysis of the Mediterranenan diet in Primary, Secondary and high school students. Rev. Child Nutr..

[50] Asensi G.D., Martínez A.S., Berruezo G.R. (2015). Cross-sectional study to evaluate the associated factors with differences between city and districts secondary school students of the southeast of Spain (Murcia) for their adherence to the Mediterranean diet. Nutr. Hosp..

[51] Galán-López P., Gisladottir T., Ries F. (2020). Adherence to the Mediterranenan Diet, Motives for Physical Exercise and Body Composition in Icelandic Adolescents: The AdolesHealth Study. Retos.

[52] El Mokhatari O., Anzid K., Hilali A., Cherkaoui M., Mora-Urda A.I., Montero-López M.D., Levy-Desroches S. (2020). Impact of migration on dietary patterns and adherence to the Mediterranean diet among Northern Moroccan migrant adolescents in Madrid (Spain). Med. J. Nutr. Metab..

[53] Mitchell T.B., Steele R.G. (2018). Latent Profiles of Physical Activity and Sedentary Behavior in Elementary School-Age Youth: Associations with Health-Related Quality of Life. J. Pediatr. Psychol..

[54] Tyler E.C., Brazendale K., Hunt E., Rafferty A., Beets M.W., Weaver R.G. (2020). Physical Activity Opportunities of Low-Income Elementary School-Aged Children During the Segmented School Day. J. Sch. Health.

[55] Grasten A., Yli-Piipari S., Huhtiniemi M., Salin K., Hakonen H., Jaakkola T. (2020). A one-year-follow-up of basic Psychological need satisfactions in physical education and associated in-class and total physical activity. Eur. Phys. Educ. Rev..

[56] Groffik D., Fromel K., Badura P. (2020). Composition of weekly physical activity in adolescents by level of physical activity. BMC Public Health.

[57] Petrie K., Pope C., Powell D. (2020). Grappling with complex ideas: Physical education, physical literacy, physical activity, sport and play in one professional learning initiative. Curr. J..

[58] Reid R.E.R., Fillon A., Thivel D., Henderson M., Barnett T.A., Bigras J.L., Mathieu M.E. (2020). Can anthropometry and physical fitness testing explain physical activity levels in children and adolescents with obesity?. J. Sci. Med. Sport.

[59] Jauk E., Ehrenthal J.C. (2020). Self-Reported Levels of Personality Functioning from the Operationalized Psychodynamic Diagnosis (OPD) System and Emotional Intelligence Likely Assess the Same Latent Construct. J. Pers. Assess..

[60] Núñez M.I.G., Muñoz M.A.C. (2020). Emotional Intelligence and Personality: Prediction of the different levels of anxiety in undergraduates studying a Degree in Pre-School and Elementary Education. Electr. J. Res. Educ. Psychol..

[61] Kim J.J., Min J.Y., Min K.B., Lee T.J., Yoo S. (2018). Relationship among family environment, self-control, friendship quality, and adolescents’ smartphone addiction in South Korea: Findings from nationwide data. PLoS ONE.

[62] Braden A., Strong D., Crow S., Boutelle K. (2015). Parent Changes in Diet, Physical Activity and behaviour in Family-Based Treatment for Childhood Obesity. Clin. Pediatr..

[63] Leclerc L., Gray-Donald K., Benedetti A., Radji S., Henderson M. (2016). The Impact of Diet on Insulin Dynamics over a 2-Year Period in Children with Family History of Obesity. Horm. Res. Pediatr..

[64] Menghetti E., Strisciugulo P., Spagnolo A., Carlettí M., Paciotti G., Muzzi G., Beltemacchi M., Concolino D., Strambi M., Rosano A. (2015). Hypertension and obesity in Italian school Children: The Role of diet, lifestyle and family history. Nutr. Metab. Cardio Dis..

[65] Dinkel D., Dev D., Guo Y.G., Sedani A., Hulse E., Rida Z., Abel K. (2020). Comparison of Urban and Rural Physical Activity and Outdoor Play Environments of Childcare Center and Family Childcare Homes. Fam. Commun. Health.

[66] Langlois J., Omorou A.Y., Vuillemin A., Briancon S., Lecomte E. (2017). Association of socioeconomic, school-related and family factors and physical activity and sedentary behaviour among adolescents: Multilevel analysis of the PRALIMAP trial inclusion data. BMC Public Health.

[67] Yang-Huang J., van Grieken A., Wang L., Jansen W., Raat H. (2020). Clustering of Sedentary Behaviours, Physical Activity, and Energy-Dense Food Intake in Six-Year-Old Children: Associations with Family Socioeconomic Status. Nutrients.

[68] Yoong S.L., Lum M., Jones J., Kerr E., Falkiner M., Delaney T., McCrabb S., Chai L.K., Seward K., Grady A. (2020). A systematic review of interventions to improve the dietary intake, physical activity and weight status of children attending family day care services. Public Health Nutr..

[69] Szczesniak M., Tulecka M. (2020). Family Functioning and Life Satisfaction: The Mediatory Role of Emotional Intelligence. Psychol. Res. Behav. Manag..

[70] Trigueros R., Navarro N., Cangas A.J., Mercaderm I., Aguilar-Parra J.M., González-Santos J., González-Bernal J.J., Soto-Camara R. (2020). The Protective Role of Emtoional Intelligence in Self-Stigma and Emotional Exhaustion of Family Members of People with Mental Disorders. Sustainability.

[71] Weinzimmer L.G., Baumann H.M., Gullifor D.P., Kouboca V. (2017). Emotional intelligence and job performance: The mediating role of work-family balance. J. Soc. Psychol..

[72] Deutsch J., Mahoney S., Waldera R., Schnabel E. (2020). The Effects of the Physical Best Health-Related Fitness Curriculum on Physical Activity Levels on Elementary Physical Education Students. Res. Q. Exerc. Sport.

[73] Kuritz A., Mall C., Schnitzius M., Mess F. (2020). Physical Activity and Sedentary Behavior of Children in Afterschool Programs: AN Accelerometer-Based Analysis in Full-Day and Half-Day Elementary Schools in Germany. Front. Public Health.

[74] Gaylis J.B., Levy S.S., Kviatkovsky S., Dehamer R., Hong M.Y. (2017). Relationships between Physical Activity, Food Choices, Gender, and BMI in Southern California Teenagers. Int. J. Adolesc. Med. Health.

[75] Labrador R.M.S. (2018). Mediterranenan Diet in Teenagers: Relation to their gender, place of residence, physical activity level and self-perceived health. Nutr. Clín. Diet. Hosp..

[76] Bonaccorsi G., Furlan F., Scouzza M., Lorini C. (2020). Adherence to Mediterranean Diet among Students from Primary and Middle School in the Province of Taranto, 2016–2018. Int. J. Environ. Res. Public Health.

[77] Kovari M., Panagiotakos D.B., Chrysohoou C., Georgousopoulou E., Tousoulis D., Pitsavos C. (2018). Gender-specific effect of Mediterranenan diet on cardiovascular disease risk; the clustering of MedDietScore components in apparently healthy males and females; 10-year follow-up of the ATTICA study. Eur. Heart J..

[78] Bliton C.F., Wolford-Clevenger C., Zapor H., Elmquist J., Brem M.J., Shorey R.C., Stuart G.L. (2016). Emotion Dysregulation, Gender, and Intimidate Partner Violence Perpetration: An Exploratory Study in College Students. J. Fam. Viol..

[79] Miller S.J. (2019). The Impact and Role of Emotions in Schools for Teachers and Students with Complex Gender Identities. Teach. Coll. Rec..

[80] Coen S.E., Davidson J., Rosenberg M.W. (2019). Towards a critical geography of physical activity: Emotions and the gendered boundary-making of an everyday exercise environment. Trans. Inst. Br. Geograph..

[81] Han K.T. (2020). The effect of environmental factors and physical activity on emotions and attention while walking and jogging. J. Leis. Res..

[82] Kruk M., Zarychta K., Horodyska K., Boberska M., Scholz U., Radtke T., Luszczynska A. (2019). What comes first, negative emotions, positive emotions, or moderate-to-vigorous physical activity?. Mental Health Phys. Act..

[83] Barnes M.J., Nagaraj P.K., Knight T.J., Jester T.W. (2019). Relations between physical activity, diet, and body composition in pediatric patients with inflammatory bowel disease. Inflamm. Bowel Dis..

[84] Laxmaiah A., Balaskrinhna N., Arlappa N., Meshran I., Harikumar R. (2017). Body Mass Index Trajectories of Indigenous Indian Adult Population and in Relation to Diet, Physical Activity and Socieconomic factors. Ann. Nutr. Metab..

[85] Hardman R.J., Meyer D., Kennedy G., Macpherson H., Scholey A.B., Pipingas A. (2020). Findings of a Pilot Study Investigating the Effects of Mediterranean Diet and Aerobic Exercise on Cognition in Cognitively Healthy Older People Living Independently within Aged-Care Facilities: The Lifestyle Intervention in Independent Living Aged Care (LIILAC) Study. Curr. Dev. Nutr..

[86] Jospe M.R., Roy M., Brown R.C., Haszard J.J., Meredith-Jones K., Fangupo L.J., Osborne H., Fleming E.A., Taylor R.W. (2020). Intermittent fasting, Paleolithic, or Mediterranean diets in the real world: Exploratory secondary analyses of a weight-loss trial that included choice of diet and exercise. Am. J. Clin. Nutr..

[87] Chang J., Morrison A.M., Lin S.H.H., Ho C.Y. (2020). How do food consumption motivations and emotions affect the experiential values and well-being of foodies?. Br. Food J..

[88] Jin H., Lin Z.B., McLeay F. (2020). Negative emotions, positive actions: Food safety and consumer intentions to purchase ethical food in China. Food Q. Ref..

[89] Soriano E., Amutio A., Franco C., Mañas I. (2020). Promoting a Healthy Lifestyle through Mindfulness in University Students: A Randomized Controlled Trial. Nutrients.

